# Clinical and genetic study of 20 patients from China with Cornelia de Lange syndrome

**DOI:** 10.1186/s12887-018-1004-3

**Published:** 2018-02-16

**Authors:** Mingyan Hei, Xiangyu Gao, Lingqian Wu

**Affiliations:** 1 Department of Pediatrics, the Third Xiangya Hospital of Central South University, Togzipo Road 138, Yuelu District, Changsha, Hunan 410013 China; 2 Neonatal Center, Beijing Children’s Hospital of Capital Medical University, Beijing, 100045 China; 3 Department of Pediatrics, Xuzhou Affiliated Hospital of East West University, Xuzhou, Jiangsu 220018 China; 4 National Key Laboratory of Medical Genetics of Central South University, Changsha, Hunan 410008 China

**Keywords:** Clinical, Genetic, Cornelia de Lange syndrome, China, Child, Newborn

## Abstract

**Background:**

Cornelia de Lange syndrome (CdLS) is a rare congenital syndrome with no racial difference. The objective of this study is to report the clinical characteristics and genetic study of 20 CdLS cases from China.

**Methods:**

This is an observational study. Suspected patients were referred for further confirmation, clinical treatment, and genetic testing under voluntary condition. Demographic data and family history, data of clinical manifestations including facial dysmorphism and developmental delay of each patient were collected. Chromosomal analysis and *NIPBL/SMC1A/SMC3* gene mutational analysis were carried out by PCR, reverse transcription PCR direct sequencing in the probands, and SNP array to detect the genome-wide copy number variations.

**Results:**

Twenty CdLS cases from China were included in this study. Facial dysmorphisms, feeding difficulties, and developmental delay were the major clinical manifestations. Seven patients underwent gene mutation tests. Both the *SMC1A* and *SMC3* gene mutation tests were negative in all. A heterozygous mutation in exon 20 of the *NIPBL* gene in proband 2, and a heterozygous mutation in intron 38 of the *NIPBL* gene in proband 3 were found in 1 patient, and RT-PCR revealed a splicing mutation in exon 38, generating both normal transcript and an aberrant alternatively spliced transcript with exon 38 deletion.

**Conclusions:**

Clinical manifestations of CdLS patients from China are similar to those in the other countries. Heterozygous mutations of *NIPBL* gene were found.

## Background

Cornelia de Lange syndrome (CdLS, OMIM#122470, 300,590, 610,759) is a rare congenital syndrome with an incidence of 0.6/100,000 birth according to data from USA [1] and 1.6–2.2/100,000 birth according to data from Europe [2]. According to reports from North America [3–5], Europe [6], and Asia [7, 8], clinical features of CdLS are facial dysmorphism +/− other organ congenital malformations, growth and developmental delay, behavioral disorders. But there is no racial difference for CdLS, It was reported that less than one tenth of CdLS patients were diagnosed within the first 28 days of life [4, 9]. The objective of this study is to report the clinical data and genetic analysis results of CdLS cases from China. This is an observational study.

## Methods

### Patient referring

Suspected patients with facial dysmorphism were referred to the Clinical Genetic Consultation Clinic of the National Key Laboratory of Medical Genetics of Central South University (for non-neonatal pediatric patients) or to Neonatal Department of the Third Xiangya Hospital of Central South University or Xuzhou Affiliated Hospital of East South University (for neonatal patients). Gene mutation tests were completed at the National Key Lab of Medical Genetics of Central South University. Pediatricians were responsible for the clinical management and treatment, genetic consultants were responsible for the family history collection and genetic laboratory examination of the patients. The patient referring and genetic testing were all under voluntary condition.

### Clinical diagnostic criteria

Diagnostic criteria of CdLS in this study are [3]: (a) Positive mutation on CdLS gene testing; or (b) Facial findings and criteria from two of the growth, development or behavior categories; or (c) Facial findings and criteria for three other categories, including one from growth, development or behavior, and two from the other categories.

### Clinical data and genetic study

The family history, demographic data (gender, delivery pattern, birth weight, patient’s age at diagnosis, maternal age/health status), and clinical data (facial characteristics, other organ congenital malformations, hypoacusis, gastrointestinal complications, mental retardation and behaviour disorders) of each patient were collected. Chromosomal analysis was completed on peripheral blood lymphocytes of the probands according to conventional techniques and high resolution banding analysis. Mutational analysis of the NIPBL, SMC1A, and SMC3 genes were carried out by polymerase chain reaction (PCR), reverse transcription PCR direct sequencing in the probands, and SNP array to detect the genome-wide copy number variations. DNA from parents was sequenced in the corresponding region when a mutation was detected in affected child.

### Ethical approval and consent

This study was conducted in accordance with the 1964 Helsinki Declaration or comparable standards, and got an ethical approval from the Institutional Review Board of The Third Xiangya Hospital of Central South University (No. 2011-S096). We obtained written consent from parents of all CdLS cases in this study for the publication of their information for research purpose.

## Results

### Demographic data

Totally 20 patients were included in this study. The demographic data was summarized in Table 1. The male to female ratio was 7:13. The average gestational age was 35 (range, 33~ 40) weeks, and the average birth weight was (2091±465) g. The average maternal age at conception was (30±4) years. The median age at diagnosis was 17 months (range from newborn to 72 months of age). There was no parental consanguinity or positive family history in any of the cases.

**Table 1:** Demographic data (Total *n* = 20)

Demographics	n	Percentage
Gender		
Male	7	35
Female	13	65
Delivery pattern		
Spontaneous vaginal delivery	15	75
Caesarian section	5	25
Low Birth Weight		
< 10th centile	18	90
< 3rd centile	14	70
Age at diagnosis		
Newborn	9	45
1–3 years	5	25
4–6 years	6	30
Maternal health status		
Healthy	17	85
Respiratory infection before delivery	1	5
Hypertension	1	5
Unilateral hydronephrosis	1	5

### Clinical manifestations

**Fig. 1:**
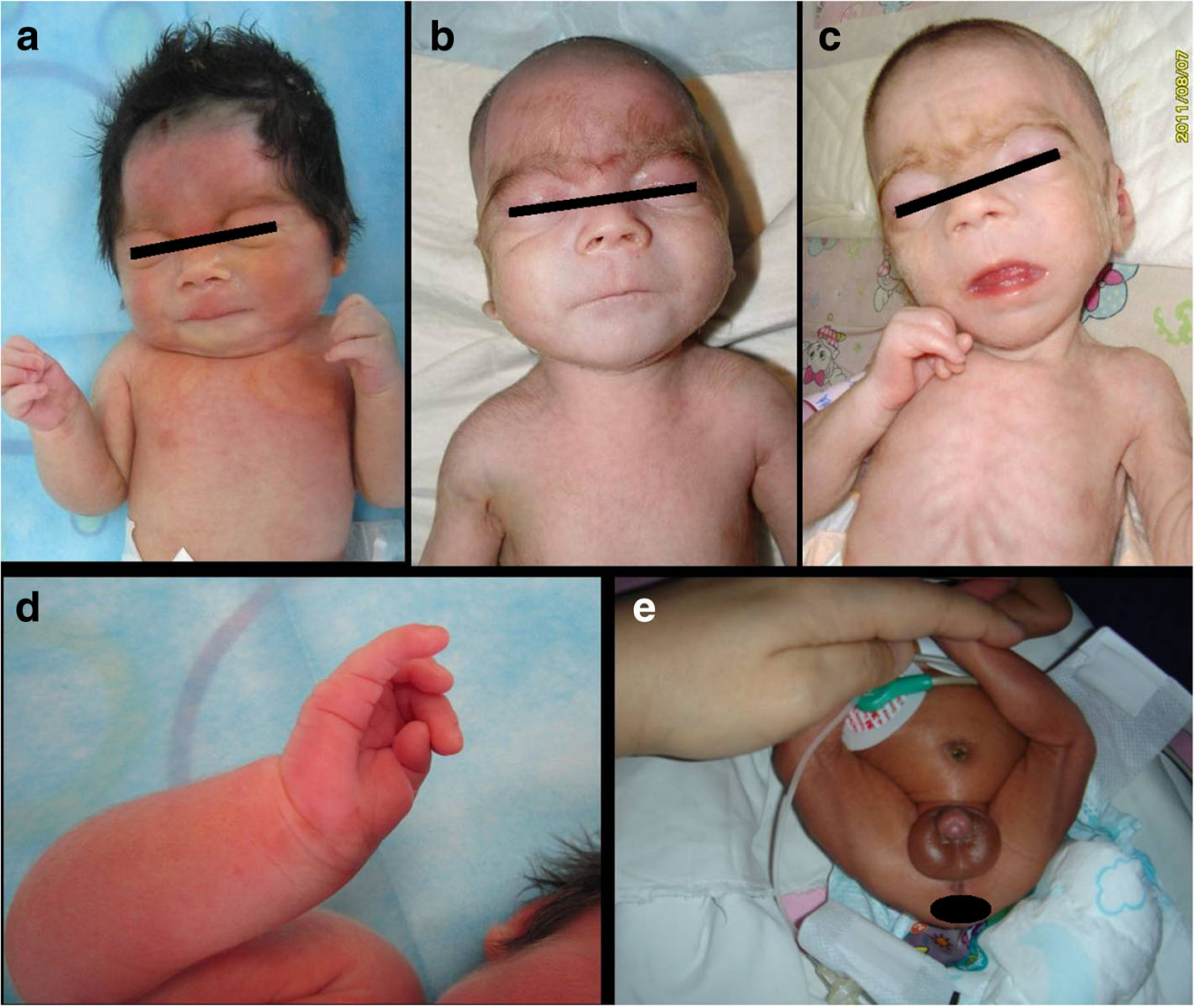
Facial and Other Dysmorphisms of 3 Chinese Cornelia de Lange Syndrome Neonates. All three neonates (**a**, **b**, **c**) had hypertrichosis of the eyebrows, synophrys, long eyelashes, broad depressed nasal bridge, and long and shallow philtrum (in neonate b and c, the excessive hair had been shaved by the parents). The marble-like skin was recognized on the chest wall. One of them was a preterm (**a**), while the other two (**b**, **c**) were term infants. Hands of the first infant were typically small with thin finger tips (**d**). The third infant had hypospadias (**e**)

Facial characteristics and clinical symptoms of the 20 CdLS cases are listed in Table 2. All patients had refractory vomiting and feeding difficulty. The echocardiography findings, karyotyping, and the extremity, heart and genital anomalies of them are listed in Table 3. 2 of the 20 patients have 2 and 3 toe syndactyly. Dysmorphic appearance of neonatal cases includes typical hypertrichosis of the eyebrows, synophrys, long eyelashes, broad depressed nasal bridge, and long and shallow philtrum, and marble-like skin (Fig. 1). All newborn patients have feeding difficulties, gastric retention and regurgitation. Only 2 out of 9 patients who were diagnosed in neonatal period completed follow up study to 4 months old due to parents’ repulse of the hospital follow-up arrangement.

**Table 2:** Selected clinical data in CdLS patients from China (*n* = 20)

Anomaly findings	No. of cases	Percentage
Facial anomalies		
synophrys	18	90.0
hypertrichosis of the eyebrows	18	90.0
long eyelashes	17	85.0
hirsutism	16	80.0
high arched palate	13	65.0
low scalp hairline	13	65.0
thin lips with down-turned corners	13	65.0
lowset ears	11	55.0
Broad, depressed nasal bridge	11	55.0
long shallow and prominent philtrum	11	55.0
Bone anomalies		
small hands with short and thin finger tips	17	85.0
hypophalangism	17	85.0
microsomy	16	80.0
the 5th finger clinodactyly	13	65.0
Other anomalies		
simian line on palms	13	65.0
genital anomaly	12	60.0
congenital heart anomaly	11	55.0
cutis marmorata	10	50.0
Clinical symptoms		
Refractory vomiting	20	100
Feeding difficulty	20	100
Developmental retardation	11	55.0
Loss of the development follow up^a^	8	40.0
Increased muscle tone	9	45.0
Decreased muscle tone	2	10.0

^a^The parents did not contact the hospital and did not answer any phone call from the hospital for unknown reason. In China, parents are paying all the Out-Patient-Department medical bills of their infants. Hence, the high rate of loss of follow-up is always a big issue in China

**Table 3:** Skeletal, heart and genital abnormalities in CdLS patients (Total *n* = 20)

No.	Gender	Karyotype	Extremity bones	ECHO	Genitals
1	Male	46, XY	No fourth finger on both hands	Normal	Bilateral crytorchidism & micropenis
2	Female	46, XX	Normal	Normal	Normal
3	Female	46, XX	Phalanx deletion of the fifth finger of both hands.	Normal	Normal
4	Male	46, XY	Normal	Normal	Right crytorchidism.
5	Female	46, XX	Phalanx deletion of the fifth finger of both hands.	Dilation of pulmonary artery.	Normal
6	Male	46, XY	Phalanx deletion of the fifth finger of both hands. Syndactyly of the second and third toes of both feet.	Normal	Normal
7	Male	46, XY	Normal	Normal	Bilateral crytorchidism.
8	Female	46, XX	Normal	Normal	Normal
9	Female	46, XX	No fourth finger on right hand. Phalanx deletion of the fifth finger of both hands.	VSD	Normal
10	Male	46, XY	Incurvation of the fifth finger of both hands. Syndactyly of the second and third toes of right foot.	Normal	Bilateral crytorchidism.
11	Female	46, XX	Normal	VSD,ASD	Gynandromorphous genitals.
12	Male	46, XY	No fourth finger on right hand.	Patent oval foramen (3 mm)	Bilateral crytorchidism. Hypospadias
13	Male	46, XY	Normal	Tiny arteriovenous fistula.	Uneven testicle size.
14	Female	46, XX	Normal	Normal	Normal
15	Female	46, XX	Phalanx deletion of the fifth finger of both	Patent oval foramen	Normal
16	Female	46, XX	Normal.	Patent oval foramen	Normal
17	Female	46, XX	Phalanx deletion of the fifth finger of both hands.	PDA (1.5 mm)	Immature
18	Female	46, XX	Phalanx deletion of the fifth finger of both hands.	Patent oval foramen, PDA (1.7 mm)	Normal
19	Male	46, XY	Normal	Normal	Normal
20	Male	46, XY	Phalanx deletion of the fifth finger of both hands.	Normal	hypospadias

*Abbreviations*: *VSD* Ventricular septum defect, *ASD* Atrial septum defect, *PDA* Patent ductus arteriosus

### Gene mutation study

The karyotyping was completed in 17 put of 19 patients (85%). There was no abnormal karyotyping finding. 13 out 20 patients’ parents rejected genetic study due to concerns of financial issues or long-term neurological problems. 7 out of 20 patients (35%) have completed NIPBL, SMC1A, and SMC3 gene mutation tests of pathogenic gene copy number variation in SNP array analysis. Positive molecular CdLS confirmation was found in 4 patients as: (1) Both the SMC1A and SMC3 genetic tests were negative in all; (2) In one patient (patient No.15 in Table 3), a heterozygous mutation (c.432 1G > T) in exon 20 of the NIPBL gene in proband 2, and a heterozygous mutation (c.6589 + 5G > C) in intron 38 of the NIPBL gene in proband 3 were found. (3) RT-PCR revealed a splicing mutation in exon 38, generating both normal transcript and an aberrant alternatively spliced transcript with exon 38 deletion. Detail information of the molecular study of these 4 patients has been published elsewhere in 2012 [10].

## Discussion

CdLS is a rare disease that occurs sporadically and is dominant paternal transmission [11] with no racial differences. Clinically, CdLS is divided into two subtypes: classic type and mild type, both having specific facial dysmorphism [12]. A population-based epidemiology study of the classic CdLS using the European Surveillance of Congenital Anomalies (EUROCAT) database established a prevalence for the classic form CdLS to be 1.24/100,000 births and the overall CdLS prevalence to be 1.6–2.2/100,000 births [2]. The antenatal diagnosis of CdLS is not always possible. However, a decreased Pregnancy-Associated Plasma Protein level in the first trimester [13] and second trimetster [14] might suggest CdLS. Schrier et al. [4] reviewed 426 CdLS cases published from 1965 to 2007 and found that only 30 (7%) were neonates. But in the present study, 9 out of 20 cases (45%) were neonates, which is much higher than that in USA. This difference of percentage of neonatal cases between China and USA is unknown. The diagnosis of 20 CdLS patients in the present study was based on the characteristic facial dysmorphisms as clinicians did in the other countries [3, 6, 15]. Kline et al. reported that dysmorphisms of CdLS patients include, in sequence, thick and long eyelashes (99%), synophrys joining at the midline and extending down to the bridge of the nose with an arched appearance of the eyebrows (98%), long prominent philtrum with down-turned lip corners (94%), small hands and feet with thin tips (90%), short and flattened nose (85%), hirsute forehead (78%), and cutis marmorata (74%). Most of these findings were observed in the Chinese CdLS patients as well and almost in the same sequence. Feeding difficulties and gastrointestinal reflux, the most important diagnostic criteria of CdLS, was observed in the neonatal patients in this study. Feeding difficulty has also been reported in earlier studies [3, 5, 11], mainly because of the refractory gastrointestinal regurgitation. A Canada study consisting of 120 CdLS children [4] reported multiple eye problems, such as ptosis iridis (44%), epiphora (22%), nasolacrimal duct obstruction (16%), blepharitis (25%), and myopia (58%). Unfortunately, the ophthalmologic evaluations were unable to be obtained for the 20 CdLS patients from China.

The etiology of CdLS is gene mutation. About 25–60% cases of CdLS are caused by point mutations in one of four genes building the cohesin system, mainly in NIPBL, and less frequently in SMC1, SMC3 and HDAC8. The three genes recognized to cause CdLS include the NIPBL gene on chromosome 5 (approximately 50% of CdLS patients carry this gene mutation) [15, 16], SMC1A gene on chromosome X (approximately 5% of CdLS patients) [17], SMC3 gene on chromosome 10 (there has been only 1 case report of this gene mutation) [18], and RAD21 and HDAC8 mutations as well [19]. Both SMC1A and SMC3 gene mutations are associated with the mild type of CdLS [15, 18]. In the Chinese cases described above, NIPBL gene mutations were also identified. Baynam et al. [20] reported an 8p23.1 deletion resulting in features of CdLS and diaphragmatic hernia, and proposed that TANKYRASE 1, a gene involved with sister chromatin cohesion from within the deleted segment, might be a novel candidate gene causing CdLS. Hayashi et al. [8] reported a 2-year-old Japanese girl with CdLS who had a balanced translocation of chromosome 12 and 13 and a 46, XX, t (5; 13) (p13.1; q12.1) karyotype. In their study, fluorescence in situ hybridization confirmed the breakpoint within NIPBL at 5p13.1, and array-based comparative genomic hybridization (array-CGH) demonstrated a cryptic 1-Mb deletion harboring six known genes at 1q25–q31.1. In the 20 Chinese cases described above, karyotyping was completed in 17 patients, but no abnormality was identified.

The intellectual disability in CdLS patients may be associated with altered gene expression as well [19]. Schrier et al. [4] reported that 63% of the CdLS patients in the United States had a birth weight less than 5th centile. In the present study, 90% of the Chinese CdLS patients were born with birth weight less than 10th centile and 70% were less than 3rd centile, and 55% of the Chinese CdLS patients had developmental retardation.

The limitations of the present study are the small number of diagnosed patients and the information of genetic study. In addition, the withdrawal of care due to the concerning of parents for the economical burden and the patients’ long-term developmental deficits is also a significant issue in China, as in China, it is the parents but not the doctors who have the legal power to decide whether a child will be taken to see a doctor and to receive medical examination or treatment. But we believe that with the development of medicine in China, more CdLS patients will be diagnosed and more genetic information will be collected in the coming future.

## Conclusions

The clinical manifestations of CdLS from China are similar to those in the other countries. Heterozygous mutations of NIPBL gene were found. Considering the small number of CdLS patients reported from China, there is a need to establish a systematic research for this disease. We hope this report will promote the recognition and attention of CdLS in China and contribute to the worldwide CdLS database.

## Abbreviations

CdLS: Cornelia de Lange syndrome
CGH: Comparative genomic hybridization
PCR: Polymerase chain reaction
PDA: Patent ductus arteriosus
VSD: Ventricular Septum Defect

## References

[1] Liu J, Baynam G (2010). Cornelia de Lange syndrome. Adv Exp Med Biol.

[2] Barisic I, Tokic V, Loane M, Bianchi F, Calzolari E, Garne E (2008). Descriptive epidemiology of Cornelia de Lange syndrome in Europe. Am J Genet A.

[3] Kline AD, Krantz ID, Sommer A, Kliewer M, Jackson LG, DR FP, Levin AV (2007). Cornelia de Lange syndrome: clinical review, diagnostic and scoring systems, and anticipatory guidance. Am J Med Genet A.

[4] Schrier SA, Sherer I, Deardorff MA, Clark D, Audette L, Gillis L (2011). Causes of death and autopsy findings in a large study cohort of individuals with Cornelia de Lange syndrome and review of the literature. Am J Med Genet A.

[5] Wygnanski-Jaffe T, Shin J, Perruzza E, Abdolell M, Jackson LG, Levin AV (2005). Ophthalmologic findings in the Cornelia de Lange syndrome. J AAPOS.

[6] Schoumans J, Wincent J, Barbaro M, Djureinovic T, Maguire P, Forsberg L (2007). Comprehensive mutational analysis of a cohort of Swedish Cornelia de Lange syndrome patients. Eur J Hum Genet.

[7] Kalal GI, Raina VP, Nayak VS, Teotia P, Gupta BV (2009). Cornelia de Lange syndrome: a case study. Genet Test Mol Biomarkers.

[8] Hayashi S, Ono M, Makita Y, Imoto I, Mizutani S, Inazawa J (2007). Fortuitous detection of a submicroscopic deletion at 1q25 in a girl with Cornelia-de Lange syndrome carrying t(5;13)(p13.1;q12.1) by array-based comparative genomic hybridization. Am J Med Genet A.

[9] Kline AD, Grados M, Sponseller P, Levy HP, Blagowidow N, Schoedel C (2007). Natural history of aging in Cornelia de Lange syndrome. Am J Med Genet C Semin Med Genet.

[10] Zhong Q, Liang D, Liu J, Xue J, Wu L (2012). Mutation analysis in Chinese patients with Cornelia de Lange syndrome. Genet Test Mol Biomarkers.

[11] Russell KL, Ming JE, Patel K, Jukofsky L, Magnusson M, Krantz ID (2001). Dominant paternal transmission of Cornelia de Lange syndrome: a new case and review of 25 previously reported familial recurrences. Am J Med Genet.

[12] Ireland M, Donnai D, Brachmann BJ (1993). De Lange syndrome delineation of the clinical phenotype. Am J Med Genet.

[13] Arbuzova S, Nikolenko M, Krantz D, Hallahan T, Macri J (2003). Low first-trimester pregnancy-associated plasma protein-a and Cornelia de Lange syndrome. Prenat Diagn.

[14] Aitken DA, Ireland M, Berry E, Crossley JA, Macri JN, Burn J (1999). Second-trimester pregnancy associated plasma protein-a levels are reduced in Cornelia de Lange syndrome pregnancies. Prenat Diagn.

[15] Dorsett D, Krantz ID (2009). On the molecular etiology of Cornelia de Lange syndrome. The year in human and medical genetics 2009. Ann N Y Acad Sci.

[16] Krantz ID, McCallum J, DeScipio C, Kaur M, Gillis LA, Yaeger D (2004). Cornelia de Lange syndrome is caused by mutations in NIPBL, the human homolog of Drosophila Melanogaster nipped-B. Nat Genet.

[17] Musio A, Selicorni A, Focarelli ML, Gervasini C, Milani D, Russo S (2006). X-linked Cornelia de Lange syndrome owing to SMC1L1 mutations. Nat Genet.

[18] Deardorff MA, Kaur M, Yaeger D, Rampuria A, Korolev S, Pie J (2007). Mutations in cohesin complex members SMC3 and SMC1A cause a mild variant of cornelia de Lange syndrome with predominant mental retardation. Am J Hum Genet.

[19] Parenti I, Gervasini C, Pozojevic J, Wendt KS, Watrin E, Azzollini J (2016). Expanding the clinical spectrum of the 'HDAC8-phenotype' - implications for molecular diagnostics, counseling and risk prediction. Clin Genet.

[20] Baynam G, Goldblatt J, Walpole I (2008). Deletion of 8p23.1 with features of Cornelia de Lange syndrome and congenital diaphragmatic hernia and a review of deletions of 8p23.1 to 8pter a further locus for Cornelia de Lange syndrome. Am J Med Genet Part A.

